# Navigated repetitive transcranial magnetic stimulation as preoperative assessment in patients with brain tumors

**DOI:** 10.1038/s41598-020-65944-8

**Published:** 2020-06-03

**Authors:** Kazuya Motomura, Hiroki Takeuchi, Ippei Nojima, Kosuke Aoki, Lushun Chalise, Kentaro Iijima, Toshihiko Wakabayashi, Atsushi Natsume

**Affiliations:** 1 Department of Neurosurgery, Nagoya University School of Medicine, Nagoya, Japan; 2 Department of Neurosurgery, Higashinagoya National Hospital, Nagoya, Japan; 3 Department of Physical Therapy, School of Health Sciences, Shinshu University, Nagano, Japan

**Keywords:** Cancer, CNS cancer, Neuroscience, Oncology, Surgical oncology, Neurology, Neurological disorders

## Abstract

We aimed to investigate clinical parameters that affected the results of navigated repetitive transcranial magnetic stimulation (nrTMS) language mapping by comparing the results of preoperative nrTMS language mapping with those of direct cortical stimulation (DCS) mapping. In the prospective, non-randomized study, patients had to meet all of the following inclusion criteria: the presence of left- or right-side brain tumors in the vicinity of or inside the areas anatomically associated with language functions; awake brain surgery scheduled; and age >18 years. Sixty one patients were enrolled, and this study included 42 low-grade gliomas and 19 high-grade gliomas (39 men, 22 women; mean age, 41.1 years, range 18–72 years). The tumor was located in the left and right hemisphere in 50 (82.0%) and 11 (18.0%) patients, respectively. In the 50 patients with left-side gliomas, nrTMS language mapping showed 81.6% sensitivity, 59.6% specificity, 78.5% positive predictive value, and 64.1% negative predictive value when compared with the respective DCS values for detecting language sites in all regions. We then investigated how some parameters, including age, tumor type, tumor volume, and the involvement of anatomical language-related regions, affected different subpopulations. Based on the receiver operating curve statistics, subgroup analysis showed that the non-involvement of language-related regions afforded significantly better the area under the curve (AUC) values (AUC = 0.81, 95% confidence interval (CI): 0.74–0.88) than the involvement of language-related regions (AUC = 0.58, 95% CI: 0.50–0.67; *p* < 0.0001). Our findings suggest that nrTMS language mapping could be a reliable method, particularly in obtaining responses for cases without tumor-involvement of classical perisylvian language areas.

## Introduction

The first-line treatment for low- and high-grade gliomas is extensive surgical resection^1–3^. Numerous clinical studies have demonstrated that gross total resection or maximum extent of resection (EOR), such as maximum resection, significantly increase progression-free survival (PFS) and overall survival (OS) in patients with gliomas^1,3–8^. However, gliomas often occur near or within functionally eloquent regions^9,10^, which means that tumor removal in such areas carries a potentially high risk for neurological function deficits, including motor dysfunction, language disturbance, and neurocognitive impairments. Intraoperative awake language mapping with direct cortical stimulation (DCS) is therefore advocated for the preservation of language function^11–13^.

While intraoperative language mapping is highly reliable, preoperative language evaluation can be of great value because the investigation of cortical language functions preoperatively makes brain tumor surgery safe, practical, and effective. Furthermore, the risk of postoperative language deficits significantly increases when brain tumor surgery involves the language dominant hemisphere; it is therefore crucial to determine language dominance for surgical planning^14,15^. Language task-based functional magnetic resonance imaging (fMRI) allows preoperative non-invasive identification of language areas. This technique has been widely used in preoperative risk assessment and planning to reduce the rate of post-surgical functional impairments^16,17^. Because a fMRI activation also represents excitatory activation, which is the result of having been recruited by an upstream or downstream area, there is a fundamental problem with fMRI to predict speech localization for presurgical planning. Although one surgical study has shown fMRI for neurosurgical planning was a useful, comparing with intraoperative DCS during awake surgery^18^, most studies have not clarified the reliability of language fMRI in preoperative neurosurgical planning for patients with tumors in language-eloquent brain regions^17,19–21^.

Navigated repetitive transcranial magnetic stimulation (nrTMS) has been increasingly used for preoperative language mapping in patients with tumors in left-side perisylvian brain regions^22–25^. These studies compared preoperative nrTMS and intraoperative DCS language mapping. nrTMS in combination with picture-naming tasks has enabled the mapping of the cerebral cortex for language-eloquent regions^22–27^. However, the accuracy of preoperative language nrTMS mapping varies across cortical language regions, protocol types, and some clinical parameters^22–24,27^. Picht *et al*. reported that pre-surgical nrTMS language maps showed high sensitivity and negative predictive values, while specificity and positive predictive values were low when compared with DCS^22^. In contrast, Tarapore *et al*. found that nrTMS language mapping had high sensitivity and specificity (90% and 98%, respectively) when compared with DCS^27^. These studies reported high numbers of false-positive findings and varying degrees of specificity and positive predictive values. We suspect that the discrepancies between these studies were caused by the influence of several pathological brain conditions, such as high-grade gliomas with broad peritumoral edemas. Krieg *et al*. compared two different picture-to-trigger intervals (PTI) (0 ms and 300 ms) and concluded that nrTMS stimulation onset coincident with picture presentation onset showed more language-involved regions than intraoperative DCS, thereby demonstrating the influence of stimulation onset on picture-naming tasks^24^. A PTI with 0 ms delay led to 90% sensitivity, 79% specificity, a 53% positive predictive value, and a 97% negative predictive value across regions. Although some groups intend to identify the optimal timing of pulse onset and new nrTMS protocols to improve the specificity of nrTMS language mapping^24,27^, it still remains unclear whether the clinical features of patients with brain tumors influence language mapping results. Clinical factors underlying the inter-individual variability of the resting motor threshold (RMT) regarding nrTMS motor mapping have been previously shown^28^; therefore, clinical factors that potentially affect the accuracy and reliability of nrTMS for language mapping should be explored.

Low-grade gliomas of World Health Organization (WHO) grade I or II are slow-growing primary brain tumors, while high-grade gliomas, such as WHO grade III or IV, show aggressiveness and rapid infiltration in the brain. Fast-growing high-grade gliomas may cause more profound language dysfunction than slower-growing low-grade gliomas, since in the former case cerebral reorganization may not occur to a great degree when neuronal damage happens at a high rate. Tumor growth could increase the extent of language impairments due to displacement or infiltration of language-eloquent brain regions. Previous studies reported the results of preoperative nrTMS language mapping performed in a comparatively large cohort of patients with high-grade gliomas^22,24,27^. However, in a study by Krieg *et al*., which showed optimal timing of pulse onset for language mapping with nrTMS, 25 out of 32 patients (78.1%) had high-grade gliomas such as glioblastoma, anaplastic astrocytoma, and anaplastic oligodendroglioma^24^. Picht *et al*. had a majority of high-grade gliomas (16/20; 80%), in a study that compared the safety and effectiveness of preoperative nrTMS with DCS mapping during awake surgery^22^. Furthermore, Tarapore *et al*. reported nine out of 12 patients (75.0%) with high-grade gliomas in a study on language mapping with nrTMS^27^. To the best of our knowledge, there is no clinical study with a majority of low-grade gliomas comparing nrTMS language mapping to intraoperative DCS during awake surgery. As the present study included mostly low-grade gliomas (68.9%), we hypothesized that such tumor effects can affect nrTMS language mapping results, with differences in tumor grade potentially altering the accuracy of preoperative language nrTMS mapping.

Regarding nrTMS language mapping, most previous studies were conducted in patients with tumors in or close to left-sided language-eloquent regions only, not right-sided regions. There is one study using nrTMS that confirmed that the right hemisphere is involved in language reorganization in patients with left hemispheric tumors^29^. Another study using nrTMS demonstrated that lesions within the language-eloquent brain can induce plasticity as a shift of language function to the non-dominant hemisphere (right hemisphere)^30^. However, the role of the right hemisphere for language in patients with right-sided tumors has not yet been determined.

In the present study, we performed preoperative language mapping using nrTMS and awake brain surgery using DCS in patients with tumors in left- and right-side brain regions that are associated with language functions. We aimed to identify the clinical parameters that affect the results of nrTMS language mapping by prospectively comparing the results of preoperative nrTMS language mapping with DCS mapping. Furthermore, we analyzed the nrTMS language mapping data of those patients with right-sided gliomas that were not located within or adjacent to anatomically defined language-eloquent brain regions.

## Results

### Patient demographic information

**Figure 1:**
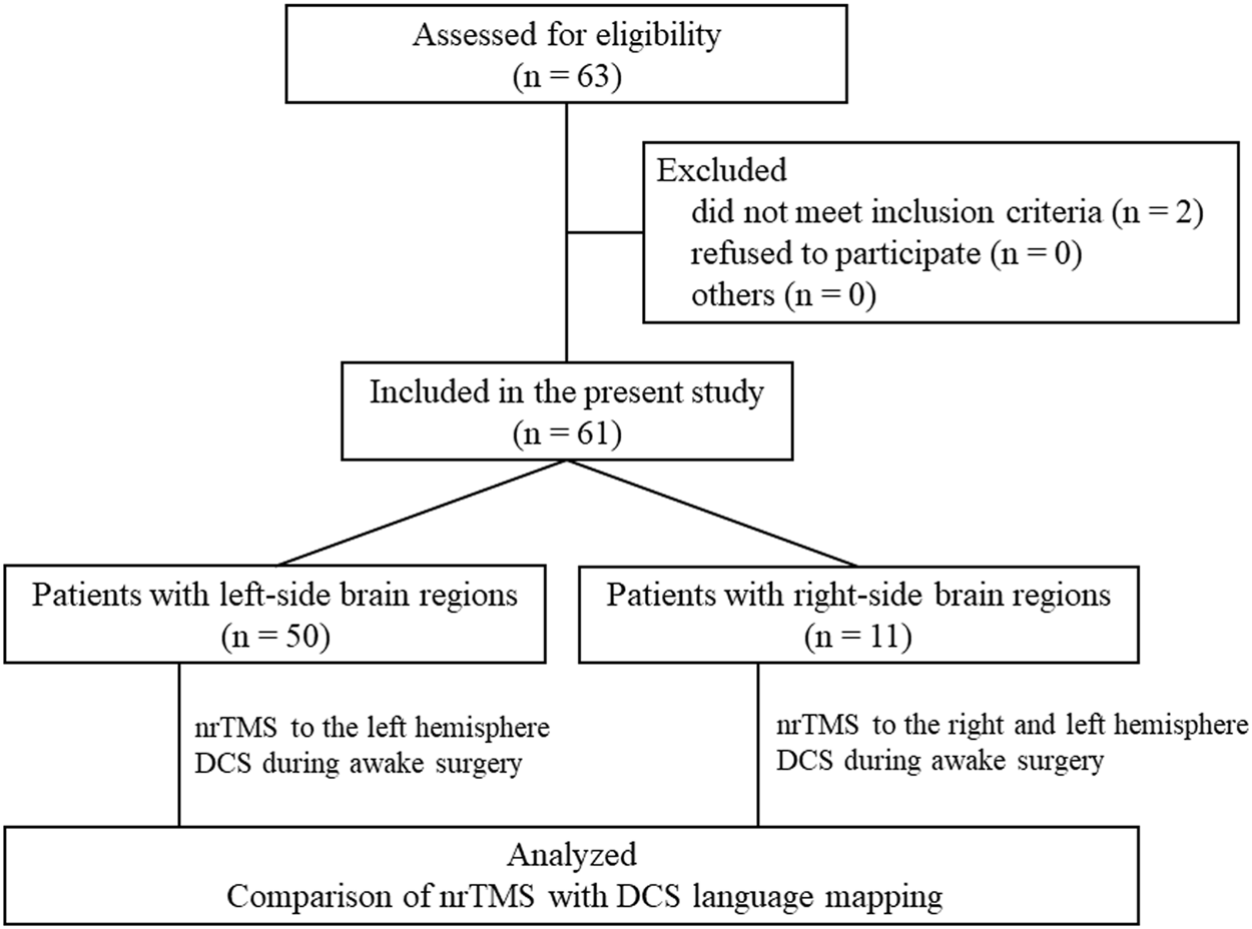
Flowchart of patient eligibility.

Between August 2013 and February 2019, 63 patients with glial tumors associated with functionally eloquent brain areas were assessed for the eligibility criteria listed above, and 61 patients (96.8%) were enrolled (Fig. 1). Clinical characteristics of all patients are summarized in Table 1. The study group consisted of 39 men and 22 women aged 18–72 years (mean age 41.1 years, median age 39 years). Sixty patients were right-handed (98.4%), and one patient was left-handed (1.6%). Histologically, this study included 42 low-grade gliomas and 19 high-grade gliomas. The tumor was located in the left and right hemisphere in 50 (82.0%) and 11 (18.0%) cases, respectively. Most of the tumors were located in the frontal lobe (n = 29, 47.5%), followed by those in the insular lobe (n = 17, 27.9%), the temporal lobe (n = 9, 14.8%), and the parietal lobe (n = 6, 9.8%). Median tumor volume, measured on MRI at preoperative diagnosis, was 43.2 cm^3^ (range, 0.6–177.3 cm^3^). The median EOR was 94.1%. A final EOR = 100% (gross total resection) was achieved in 25 patients (41.0%), an EOR ≥ 90% <100% (subtotal resection) in 16 (26.2%) patients, and an EOR < 90% in 20 (32.8%) patients.

**Table 1:** Demographics and clinical characteristics.

Parameters	No. of patients	%
	(n = 61)	
Age (years)		
mean	41.1	
median	39	
range	18–72	
Sex		
male	39	63.9
female	22	36.1
Handedness		
right-handed	60	98.4
left-handed	1	1.6
Histologic type		
pilocytic astrocytoma	1	1.6
dysembryoplastic neuroepithelial tumor (DNT)	2	3.3
pleomorphic xanthoastrocytoma (PXA)	1	1.6
diffuse astrocytoma (DA)	29	47.5
oligodendroglioma (OG)	9	14.8
anapalastic astrocytoma (AA)	10	16.4
anaplastic oligodendroglioma (AO)	5	8.2
glioblastoma (GBM)	4	6.6
Tumor type (WHO grade)		
low grade (grade I, II)	42	68.9
high grade (grade III, IV)	19	31.1
Side of lesion		
left	50	82.0
right	11	18.0
Tumor location		
frontal	29	47.5
insular mainly	17	27.9
temporal	9	14.8
parietal	6	9.8
Tumor volume (cm^3^)		
median	43.2	
range	0.6–177.3	
Final extent of resection		
Median (%)	94.1	
100% (=gross total resection)	25	41.0
>90%, <100% (=subtotal resection)	16	26.2
<90%	20	32.8

Abbreviation: insular mainly; insular + temporal and/or frontal.

### Preoperative nrTMS language mapping in patients with left-side brain tumors

Preoperative nrTMS language mapping was performed in the 50 patients with left-side brain tumors over the whole left cerebral hemisphere. While five of the 50 patients (10.0%) asked us to reduce the stimulation intensity due to discomfort, pain, or muscle tetanization related to transient temporal muscle activation, no other adverse events were observed.

The left hemisphere was stimulated at 145–522 sites (median, 422 sites). Median RMTs, as a percentage of the stimulator output, were 46% (range, 32–57%). nrTMS intensity mapping (between 60% and 100%) of each participant’s own RMT was carried out in all 50 patients. Each nrTMS train consisted of 10 pulses given at rates between 5 and 10 Hz (Table 2). Trains with 5-Hz frequency were well tolerated and used most often.

**Table 2:** Patient characteristics and nrTMS and DCS language mapping parameters used in the 50 glioma patients with left-side brain regions.

Pt No.	Age	Sex	Handedness	Tumor location	Tumor volume	Pathology	WHO grade	RMT	Hz	No. of pulses	%RMT	DCS intensity (mA)	Involvement of language regions	Preoperative language disturbance	Postoperative transient language disturbance	Postoperative permanent language disturbance
								(%)		in Train	(%)					
1	67	M	R	Insular	107.6	AA	III	57	5	10	100	4	+	+	+	+
2	64	M	R	Frontal	85.1	DA	II	50	10	10	100	4	+	−	−	−
3	41	M	R	Frontal	8.8	OA	II	55	10	10	100	3	−	−	+	−
4	63	M	R	Insular	85.1	DA	II	40	5	10	100	4	−	−	+	−
5	72	M	R	Insular	24.9	DA	II	50	5	10	80	4	−	−	+	−
6	28	M	R	Parietal	0.6	PXA	II	48	5	10	80	4	−	−	−	−
7	39	M	R	Insular	61.7	DA	II	45	5	10	80	3	−	−	−	−
8	43	M	R	Frontal	101.9	AO	III	50	5	10	100	3	+	−	+	−
9	38	F	R	Insular	85.5	DA	II	53	5	10	80	4	−	−	−	−
10	18	M	R	Insular	109.4	GBM	IV	45	5	10	100	4	+	+	+	+
11	29	M	R	Insular	45.3	DA	II	40	7	10	80	4	−	−	+	−
12	44	M	R	Frontal	14.7	DA	II	46	5	10	100	4	−	−	−	−
13	46	M	R	Temporal	25.7	GBM	IV	50	5	10	80	3	+	−	−	−
14	59	M	R	Insular	91.8	AO	III	32	5	10	100	2.5	−	−	−	−
15	33	M	R	Insular	12.2	OG	II	50	5	10	100	3	−	−	+	−
16	30	F	R	Frontal	18.8	DA	II	35	5	10	100	3	−	−	−	−
17	57	M	R	Frontal	105.3	DA	II	38	5	10	100	3	+	−	−	−
18	20	M	R	Frontal	19.7	DNT	I	43	5	10	100	3	−	−	+	−
19	27	M	R	Frontal	50.2	DA	II	55	5	10	60	3	−	−	+	−
20	44	M	R	Frontal	39.7	DA	II	45	5	10	80	3	−	−	−	−
21	42	M	R	Parietal	86	DA	II	45	5	10	80	3	+	−	+	+
22	67	M	R	Temporal	79.3	AA	III	50	5	10	80	8	+	+	+	+
23	31	F	R	Temporal	57.8	AA	III	50	5	10	80	6	+	−	−	−
24	45	M	R	Temporal	27.5	AA	III	42	5	10	80	4	+	−	−	−
25	60	F	R	Parietal	38.3	AO	III	45	5	10	80	3	+	−	+	−
26	47	F	R	Temporal	29.2	DA	II	42	5	10	80	3	+	−	−	−
27	29	M	R	Frontal	10.6	AA	III	40	5	10	80	3	−	−	−	−
28	45	M	R	Frontal	51.3	DA	II	55	5	10	80	3	−	−	+	−
29	40	F	R	Frontal	8.7	OG	II	45	5	10	80	3	−	−	−	−
30	23	F	R	Frontal	41.1	DNT	I	48	5	10	80	3	−	−	−	−
31	20	M	R	Temporal	22.4	Pilo	I	40	5	10	80	4	+	−	−	−
32	33	F	R	Frontal	50.1	DA	II	50	5	10	80	3	−	−	−	−
33	23	F	R	Temporal	14.4	AA	III	36	5	10	100	2.5	−	−	−	−
34	35	F	R	Frontal	62.2	OG	II	40	5	10	80	1.5	+	−	+	−
35	53	M	R	Insular	55.1	DA	II	46	5	10	80	1.5	−	−	+	−
36	18	M	R	Frontal	17.8	DA	II	46	5	10	80	3	−	−	−	−
37	34	M	R	Temporal	22.4	DA	II	45	5	10	80	3	+	−	+	−
38	67	F	R	Insular	40.1	DA	II	45	5	10	80	3	−	−	+	−
39	43	M	R	Frontal	9	AA	III	48	5	10	80	3	+	−	+	−
40	27	F	R	Insular	177.3	DA	II	48	5	10	80	3	+	−	+	−
41	26	M	R	Frontal	30.7	AA	III	47	5	10	80	3	−	−	−	−
42	29	F	R	Temporal	69.6	AA	III	47	5	10	80	3	+	−	−	−
43	34	F	R	Parietal	43.2	OG	II	41	5	10	80	3	+	−	+	−
44	31	M	R	Frontal	9.4	DA	II	46	5	10	80	3	−	−	−	−
45	68	M	R	Frontal	12.6	AO	III	40	5	10	80	2.5	+	−	−	−
46	58	F	R	Frontal	8.4	DA	II	40	5	10	80	2.5	+	−	−	−
47	36	M	R	Frontal	84.2	OG	II	48	5	10	60	2.5	−	−	−	−
48	38	F	R	Parietal	14.2	DA	II	45	5	10	60	1.5	−	−	−	−
49	60	F	R	Frontal	111.5	AA	III	48	5	10	80	2	+	−	−	−
50	28	F	R	Frontal	120.5	AO	III	46	5	10	80	2.5	−	−	−	−

Abbreviation: nrTMS; navigated repetitive transcranial magnetic stimulation, DCS; direct cortical stimulation, RMT; resting motor threshold; %RMT; stimulation intensity in percentage resting motor threshold, Pilo; pilocytic astrocytoma, DNT; dysembryoplastic neuroepithelial tumor, PXA; pleomorphic xanthoastrocytoma, DA; diffuse astrocytoma, OG; oligodendroglioma, AA; anapalastic astrocytoma, AO; anaplastic oligodendroglioma, GBM; glioblastoma.

Twenty-two patients (44.0%) showed involvement of anatomical language regions inside the brain tumors. During the preoperative course, three patients (6.0%) had already exhibited mild language disturbances. After tumor resection with awake brain mapping, 21 of all 50 patients with left-side brain tumors (42.0%) developed transient speech disorders and four (8.0%) exhibited permanent language disorders.

### Accuracy of nrTMS language mapping in patients with left-side gliomas

Awake brain surgery was successfully performed with intraoperative functional mapping using DCS in all 50 patients with left gliomas. The minimum DCS intensities used in positive language site are shown in Table 2. In these patients, we compared the results of nrTMS and DCS language mapping in 278 regions according to Corina’s cortical parcellation system (CPS). Responses of intraoperative DCS mapping were positive for 1–8 areas (median, 3 areas), whereas responses of nrTMS were positive for 0–8 regions (median, 4 regions). There was no significant difference in the mean DCS intensities between the regions negative with nrTMS and positive with DCS (3.36 mA) and the regions negative with nrTMS and negative with DCS (3.31 mA).The overall accuracy values (sensitivity, specificity, positive and negative predictive values) for all regions in the 50 patients with left-side gliomas are shown in Table 3. Sensitivity was 81.6%, specificity was 59.6%, the positive predictive value was 78.5%, and the negative predictive value was 64.1%, for detecting language sites across all regions. Sensitivity (89.5%) and the positive predictive value (82.8%) were higher in the anterior compared with the posterior language-related CPS regions, while specificity (73.5%) and the negative predictive value (71.4%) were higher in the posterior compared with the anterior language-related CPS regions.

**Table 3:** Sensitivity, specificity and positive/negative predictive values for all brain regions, anterior and posterior language-related regions in left-side and right-side brain tumor patients.

Parameters	Sensitivity (%)	Specificity (%)	Positive predictive value (%)	Negative predictive value (%)
Left-side brain tumor				
All brain regions	81.6	59.6	78.5	64.1
Anterior language-related regions	89.5	28.1	82.8	40.9
Posterior language-related regions	63.6	73.5	66.0	71.4
Right-side brain tumor				
All brain regions	66.7	100	100	98.8

Anterior language-related regions; trIFG, opIFG and vPrG, posterior language-related regions; aSMG, pSMG, anG, mSTG and pSTG.

### Effect of tumor involvement of language-related regions on nrTMS language mapping results

**Figure 2:**
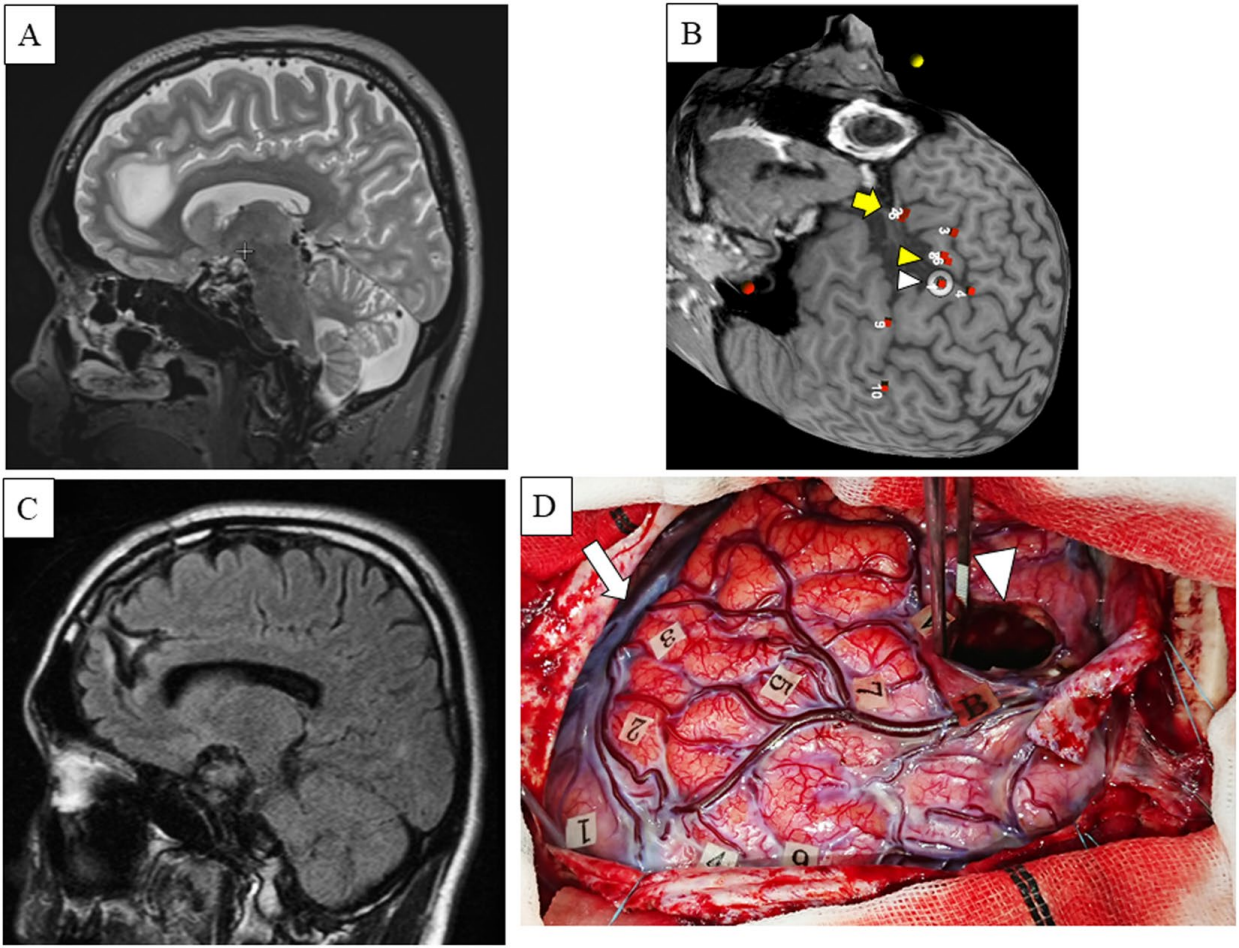
(**A**) Representative image of patient 44. Preoperative axial fluid-attenuated inversion recovery-weighted magnetic resonance images showing a left frontal low-grade glioma (left cingulate gyrus) in a 31-year-old right-handed man with no relevant medical history who presented with episodic headache. (**B**) Preoperative three-dimensional images obtained with navigated repetitive transcranial magnetic stimulation (nrTMS) language mapping using the picture-naming task. Anomia was induced in the precentral gyrus (white arrowhead) and in the opercular part of the inferior frontal gyrus (yellow arrowhead). Phonemic paraphasia was induced in the triangular part of the inferior frontal gyrus (yellow arrow). These responses correspond to the intraoperative image (Fig. 4D) showing cortical mapping with direct cortical stimulation (DCS). (**C)** Postoperative axial fluid-attenuated inversion recovery-weighted magnetic resonance imaging showing gross-total resection of the mass using awake brain mapping. (**D**) Intraoperative image obtained after resection showing letter tags that indicate tumor boundaries (**A–B**). Stimulation over the precentral gyrus induced speech arrest (number tag: 1); stimulation over the opercular part of the inferior frontal gyrus induced speech arrest (number tag: 2); stimulation over the triangular part of the inferior frontal gyrus induced semantic paraphasia (number tag: 3). The dorsolateral prefrontal cortex (DLPFC) was mapped on the lateral tumor side (number tags: 5, 7), using a 5-digit backward digit span task to confirm verbal working memory. Furthermore, positive responses were detected using a 4-digit backward digit span task (number tags: 4, 9); arrowhead, corridor; arrow, sylvian fissure.

**Figure 3:**
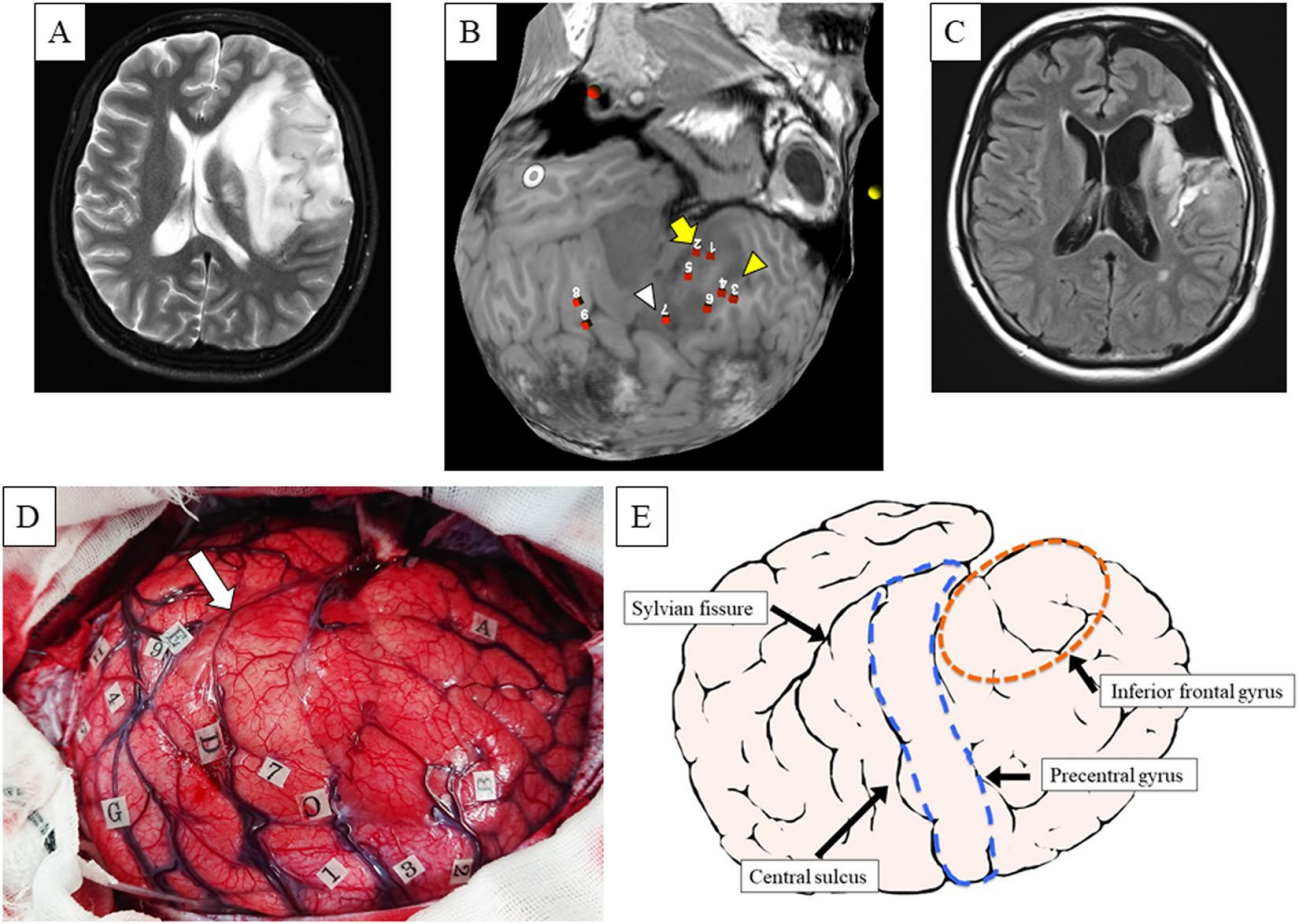
(**A**) Representative images of patient 40. Preoperative axial T2-weighted magnetic resonance images showing a high-intensity mass lesion mainly in the left insular lobe that extended widely to the left inferior and middle frontal gyrus in a 27-year-old, right-handed woman with no relevant medical history. She presented with convulsive attacks and was transferred to a nearby hospital in December 2017. She was then referred to the authors’ hospital to undergo tumor removal using awake surgery. (**B**) Preoperative three-dimensional images obtained with navigated repetitive transcranial magnetic stimulation (nrTMS) language mapping using the picture-naming task. Semantic paraphasia was induced in the triangular part of the inferior frontal gyrus (yellow arrow) and the dorsolateral prefrontal cortex (DLPFC) (yellow arrowhead). Anomia was induced in the posterior part of the middle frontal gyrus (white arrowhead). These responses are in conflict with the intraoperative image (Fig. 5D) showing cortical mapping with direct cortical stimulation (DCS). (**C**) Postoperative sagittal fluid-attenuated inversion recovery-weighted magnetic resonance imaging revealing subtotal mass resection using awake brain mapping. The patient exhibited transient postoperative language impairments and motor paralysis. (**D**) Stimulation over the precentral gyrus induced speech arrest (number tag: 1, 7); stimulation over the dorsal premotor cortex induced speech slowness (number tags: 2, 3); stimulation over the opercular and triangular part of the inferior frontal gyrus induced no speech disturbance. Using direct cortical stimulation (DCS), language disturbances were detected in the precentral gyrus in the upper part of the tumor (number tag: 7), where language functions might have been moved from the opercular and triangular part of the inferior frontal gyrus. Stimulation over the posterior portion of the superior temporal gyrus induced phonemic paraphasia (number tags: 4, 5, 9); arrow, sylvian fissure. (**E**) Schematic diagram showing the intraoperative DCS language mapping.

Next, we sought to determine how subpopulations were affected differently by different parameters, including age, tumor type, tumor volume, and the involvement of anatomical language-related regions. A subgroup analysis showed that involvement of language-related regions compared to non-involvement of language-related regions was associated with lower sensitivity (70% vs. 90.9%, respectively), specificity (46.9% vs. 72.0%, respectively), a lower positive predictive value (68.3% vs. 86.5%, respectively), and a lower negative predictive value (48.9% vs. 80.0%, respectively) (Table 4, Figs. 2 and 3). We obtained similar results for accuracy across groups for age (≥40 or <40 years), tumor type (high- or low-grade), and tumor volume (≥40 or <40 cm^3^).

**Table 4:** Sensitivity, specificity and positive/negative predictive values in the subgroup of patients according to the age, tumor type, tumor volume and involvement of language-related regions.

Parameters	N	Sensitivity (%)	Specificity (%)	Positive predictive value (%)	Negative predictive value (%)
Age (years)					
>40	24	83.3	61.8	78.1	69.4
<40	26	79.7	56.8	78.9	58.1
Tumor type					
high grade	17	80.0	58.8	78.8	60.6
low grade	33	82.4	60.0	78.3	66.1
Tumor volume (cm^3^)					
>40	26	82.8	64.2	80.2	68.0
<40	24	80.2	54.3	76.7	59.5
Involvement of language-related regions					
+	22	70	46.9	68.3	48.9
−	28	90.9	72.0	86.5	80.0

**Figure 4:**
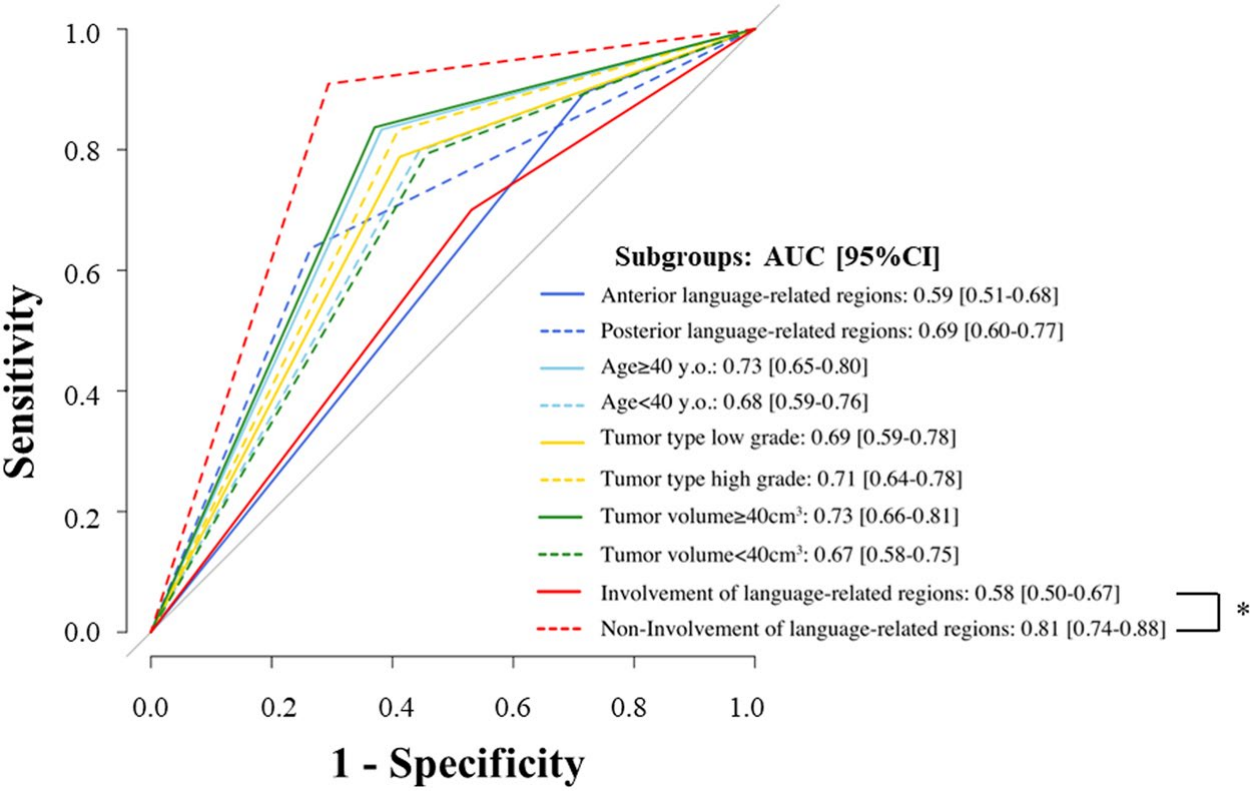
Receiver operating characteristics (ROC) curve of navigated repetitive transcranial magnetic stimulation (nrTMS) vs. direct cortical stimulation (DCS) in each subgroup. The ROC is plotted between the true-positive rate (sensitivity) on the Y-axis and the false-positive rate (1-specificity) on the X-axis. Predictive accuracy of nrTMS was evaluated using ROC analysis. The area under the ROC curve (AUC) and 95% confidence intervals (CI) are listed for each subgroup. The statistical significance of observed AUC differences between the scores was assessed using DeLong’s test^60^; *p < 0.0001.

Furthermore, we calculated receiver operating characteristic (ROC) curve statistics for the sensitivity, specificity, positive predictive values, and negative predictive values (Fig. 4). Notably, our results show that the non-involvement of language-related regions led to significantly better AUC values (AUC = 0.81, 95% CI: 0.74–0.88) than the involvement of language-related regions (AUC = 0.58, 95% CI: 0.50–0.67; *p* < 0.0001; Figs. 2–4).

### Determination of hemispheric language dominance using nrTMS language mapping

In the 11 patients with right-side brain tumors, we performed preoperative nrTMS language mapping of both the left and right cerebral cortex. As shown in Table 5, all 10 right-handed patients showed language errors in the left hemisphere and no language errors in the right hemisphere. One left-handed patient’s language area was located in the right cerebral cortex (Table 5). These findings suggest that nrTMS language mapping enables the determination of hemispheric language dominance in patients with brain tumors.

**Table 5:** Language dominancy according to the nrTMS as compared with the results of language mapping by DCS in right-side brain tumor patients.

	Language mapping by DCS	
	Positive	Negative
Language dominance by nrTMS		
Left	0	10
Right	1	0

Abbreviation: nrTMS; navigated repetitive transcranial magnetic stimulation, DCS; direct cortical stimulation.

## Discussion

### Clinical parameters associated with results of nrTMS language mapping

The purpose of our study was to investigate the impact of clinical parameters on the results of nrTMS language mapping and their reliability in relation to DCS. The effects of clinical parameters on the results of nrTMS language mapping were investigated by determining accuracy and calculating ROC curves in each subgroup; the parameters included age, tumor type, tumor volume, and the involvement of language-related regions (Table 4, Fig. 4). We detected a significant difference regarding the involvement of language- and non-language-related regions between groups (Fig. 4). These findings were considerably affected by the presence of brain lesions (Figs. 2 and 3).

A previous study has yielded controversial results on nrTMS language mapping. Ille *et al*. reported that nrTMS language mapping in 27 patients with left-side perisylvian lesions was not affected by the presence of brain lesions when comparing with intraoperative DCS language mapping^23^. There are, however, some critical differences in clinical features and methodology between our study and the aforementioned one. First, the number of low-grade gliomas in our group was larger than that of the study by Ille *et al*.^23^. Unlike high-grade gliomas, low-grade gliomas often contain several brain functions, such as motor, language, and cognitive functions, inside the tumor. Furthermore, low-grade infiltrating gliomas are characterized by progressive functional reshaping because of their slow growth even before treatment. Such functional modifications can occur during the natural history of disease. The location of lesions within language-function areas had several patterns in each patient with low-grade gliomas. We assume that functional language reorganization occurs when a tumor with low-grade glioma invades the language-related regions. Therefore, it may be difficult for nrTMS language mapping to detect scattered localized language areas inside the tumor. On the other hand, cerebral reorganization of high-grade gliomas may not be possible; thus, brain functional modification may not be induced by fast-growing lesions. Therefore, language functions may remain associated with the language-related regions regardless of tumor invasion. These differences in tumor types may explain the different results of nrTMS language mapping between the two studies. In addition, there is a difference in the picture-to-trigger interval between the two studies. The nrTMS language mapping procedure in Ille *et al*.’s study used a picture-to-trigger interval time of 300 ms for 81% of patients and 0 ms for 19% of patients. In contrast, in the current study, synchronization of the nrTMS stimulation started at 300 ms after image presentation for all patients. Since Ille *et al*.’s study included patients for whom nrTMS stimulation onset coincided with picture presentation onset, the accuracy of their study’s nrTMS language mapping procedure might have been more accurate compared with the results of the present study. This methodological difference may also account for the difference regarding tumor involvement of language-related regions during nrTMS language mapping between the two studies.

### Accuracy of nrTMS language mapping for all brain regions

nrTMS has increasingly been used as a preoperative evaluation procedure at a number of institutions, because it allows for language mapping before awake surgery with DCS^27,31^. The combination with neuro-navigation and electric fields has dramatically improved the clinical value of the TMS technique^32,33^. Some studies assessing the relationship between nrTMS and DCS reported that nrTMS is superior to other preoperative modalities, such as fMRI, electrocorticography, and magnetoencephalography^22,25–27^. Further, nrTMS yields higher overall sensitivity (>90%) and negative predictive values (>80%), compared with DCS language mapping^22–24,27,34^. One group reported high specificity (98%) and positive predictive values (69%)^27^, while other studies have shown low specificity (<25%) and positive predictive values (<40%)^22–24,34^. The former group started nrTMS language mapping at 110% of RMT using a pulse train of 10 pulses at 5 Hz^27^. In the latter groups, Picht *et al*. used three patterns: (a) 5 Hz, 5 pulses, 100% RMT; (b) 7 Hz, 7 pulses, 100% RMT; (c) 10 Hz, 7 pulses, 100% RMT^22^. In addition, the train of TMS pulses started 300 ms after the picture presentation onset. On the other hand, IIIe *et al*. reported the following stimulation parameters: three setups of nrTMS bursts (5 Hz, 5 pulses; 7 Hz, 5 pulses; 7 Hz, 7 pulses) with an intensity of 100% RMT^23^. Krieg *et al*. applied a 5-Hz pulse train starting at either 0 ms or 300 ms post picture presentation onset^24^. Median RMT was 90% at 0 ms stimulation onset and 100% at 300 ms post picture presentation onset. Compared to these studies, the current study used slightly different parameters: a rTMS train of 10 pulses at 5–10 Hz, with an RMT of 60–80% (Table 2). In addition, we employed a 300-ms delay between object presentation and nrTMS stimulation (Table 2). Regarding the tumor location, the previous studies included the frontal, temporal and parietal lobes. All previous groups had approximately 30–60% of tumors in the temporal lobe. In contrast to these groups, there were only few temporal tumors in our study (an approximate 15%) (Table 1). One possible reason for these differences is that different intervals between picture presentation and nrTMS stimulation onset were adopted between study groups. In the former study, nrTMS stimulation onset was simultaneous with picture presentation, while other groups employed a 300-ms delay between object presentation and nrTMS stimulation. Additionally, differences in results might be expected, because the language system is complex regarding cortical and subcortical language networks. nrTMS motor mapping has revealed that TMS activates motor neurons via indirect intracortical neuro-pathways, whereas DCS directly activates motor cortical fibers^35^. The unspecific activation and inhibition of intracortical neuronal pathways might render TMS stimulation spots positive that have no crucial language functions. In the current study, we obtained high positive predictive values compared to other reports, which suggests preoperative nrTMS mapping as a reliable technique for neurosurgeons (Table 3). Furthermore, our nrTMS language mapping results yielded low overall sensitivity and negative predictive values compared with prior studies (Table 3)^22–24,27^. Considering that the group with non-involvement of language-related regions showed high sensitivity and negative predictive values, it seems that the presence of brain lesions played a role in these diverging results. Moreover, it has been reported that a 5-Hz pulse train starting at 0 ms post picture presentation onset leads to high accuracy in preoperative language maps^24^. Thus, a nrTMS protocol with a picture-to-trigger interval of 0 ms might have improved the accuracy of our nrTMS language mapping results^36^.

Another reason for the differences in the accuracy of nrTMS language mapping was the use of a different nrTMS system among research groups. We have applied an image-guided rTMS using the commercial neuronavigation system Visor^2^ (ANT Neuro, Enschede, the Netherlands) and real-time visualization of the Figure 8 coil (Magstim, UK). On the other hand, most of the nrTMS system in previous studies adopted a different nrTMS system, the eXimia NBS version 3.2.2 and Nexstim NBS 4.3. with a NEXSPEECH module (Nexstim Oy, Helsinki, Finland). Using a different nrTMS system may account for the differences between previous and current results.

### nrTMS language mapping and determination of language dominance

Although some groups have sought to determine language dominance with nrTMS, results remain inconclusive. In the present study, we did not perform the intracarotid amobarbital procedure or the Wada test, which are gold standards for the lateralization of language dominance^37^. The Wada test is an invasive examination with a significant risk of neurological impairment and patient discomfort; therefore, there has been great interest in replacing it with a non-invasive procedure. Compared with intraoperative DCS during awake surgery, preoperative fMRI for language function is insufficiently accurate to serve as a basis for the decision-making of brain surgery^19–21^. However, some studies have shown that preoperative lower-frequency nrTMS predictions are well correlated with the results of DCS language mapping during awake brain surgery^22,34^. In this study, we demonstrated an excellent correlation between nrTMS-derived language laterality and the DCS procedure during awake surgery (Table 5). In addition, we were able to determine hemispheric language dominance in patients with tumors in right-side brain regions using nrTMS language mapping in the cerebral cortex.

### Limitations

Our results are somewhat limited compared to those of randomized clinical trials, because non-randomized studies may be influenced by unrecognized biases. Furthermore, the present study was based on a small number of cases. Therefore, a large randomized study is required to further establish the role of nrTMS in preoperative language mapping in patients with gliomas. Additional evidence from nrTMS language mapping studies is crucial for our understanding of preoperative language assessments before awake surgery.

Furthermore, one of the limitations of our study is the inclusion of hesitation errors in the analysis. In cases of delayed answers during stimulation, we had difficulty in differentiating “difficulty of word recall” from “hesitations” objectively. Despite the fact that the analysis was performed by experienced examiners, subjectivity cannot be ruled out. In fact, hesitations are commonly regarded as a comparatively untrustworthy error category, and, thus, they were mostly excluded. The interpretation of hesitation errors might be a cause of increased false-positive results when comparing with DCS.

## Conclusions

To investigate the clinical parameters that affect the results of nrTMS language mapping, we prospectively compared the results of preoperative nrTMS and DCS language mapping. Tumor involvement of anatomical language-related regions significantly affected the results of the nrTMS mapping. Furthermore, nrTMS language mapping enabled the determination of hemispheric language dominance in a preoperative assessment of patients with gliomas. Our findings suggest that nrTMS language mapping could be a reliable method, particularly in obtaining responses for cases without tumor-involvement of classical perisylvian language areas. Further studies are required to validate nrTMS language mapping as a preoperative mapping tool of cortical language function.

## Materials and Methods

### Study design

This clinical trial was designed as a single-center, prospective, non-randomized study to assess the clinical factors that affect the results of nrTMS language mapping when preoperative nrTMS language mapping is prospectively compared with DCS mapping. All patients were scheduled for awake brain surgery at our neurosurgical department and underwent preoperative language mapping with nrTMS while performing a picture-naming task the day before the surgery. The study protocol was approved by the Ethics Committee of Nagoya University Hospital (approval number: 2013-0094) and carried out in accordance with the Declaration of Helsinki^38^. Written informed consent was obtained from all participants included in the study.

### Patients

The process of enrollment and exclusion of participants is presented in Fig. 1. For inclusion in this study, patients had to meet all of the following criteria: the presence of left- or right-side brain tumors in the vicinity of or inside the areas anatomically associated with language functions; awake brain surgery scheduled; and age >18 years. The exclusion criteria were as follows: severe cognitive disorders; severe aphasia; psychiatric disorders and higher brain dysfunctions identified during preoperative neuropsychological testing; patients who were not able to execute one or more of three tests, including the Standard Language Test of Aphasia (SLTA)^39^, the third edition of the Wechsler Adult Intelligence Scale (WAIS-III)^40^, or the Wechsler Memory Scale-revised (WMS-R)^41^; a heart pacemaker or drug infusion pump; severe heart disease; currently undergoing deep brain or spinal cord stimulation; and pregnancy or intention to get pregnant.

Sixty-one patients who underwent preoperative language mapping using nrTMS and awake surgery with intraoperative direct electric mapping for gliomas associated with language functions were enrolled in the current study. All patients were treated at Nagoya University Hospital (Nagoya, Japan) between August 2013 and February 2019.

### Pre- and post-operative neuroradiological evaluation

Preoperative navigational MRI, including three-dimensional contrast T1-weighted imaging, conventional MRI (two-dimensional T1- and T2-weighted imaging), and diffusion-weighted imaging data were acquired for this study using a 3.0-Tesla scanner (Siemens MAGNETOM Verio; Siemens Healthcare, Erlangen, Germany) with a 32-channel head coil.

Our MRI protocol consisted of the following imaging parameters: whole-brain; high-resolution; T1-weighted; phase encoding direction, anterior to posterior; matrix size, 256 × 256; repetition time (TR), 2.5 s; echo time (TE), 2.48 ms; 192 slices; slice thickness, 1 mm with no gaps; flip angle, 15°; voxel size, 1 × 1 × 1 mm^3^; field of view (FOV), 256 mm. We performed the volumetric analysis to define tumor volume using the iPlan cranial planning software included in the BrainLAB iPlan Cranial version 2.6 and 3.0^42^ (German HealthCare Export Group, Bonn, Germany).

The pre- and post-operative tumor volumes were measured to estimate EOR using contrast T1-weighted when the tumor mass was enhancing, while T2-weighted MRI was used for non-enhancing tumor before and after tumor resection. The EOR was calculated by two independent experienced neurosurgeons (L.C, K.I) using the following formula (preoperative tumor volume – postoperative tumor volume)/preoperative tumor volume.

### Preoperative nrTMS language mapping

Cortical nrTMS language mapping was performed using the Magstim Rapid^2^ biphasic stimulator to generate repetitive magnetic pulses (Magstim Co. Ltd., Whitland, UK). The pulses were delivered with a standard 70-mm figure-8 coil. We co-registered three-dimensional T1-weighted MRI as an anatomic reference and the patient’s brain to observe the cortical areas during nrTMS. The nrTMS neuro-navigation system displayed the electric field induced by the stimulating coil, which is directly visualized over the three-dimensional reconstruction of the patient’s brain^43^. We used electric-field-navigated TMS, which is clinically accurate and effective in cortical mapping. The hotspot can be defined as the accurate location of the calculated strongest electric field that the navigator visualizes on the individual MRI of the cortex. This system can be used to visualize the real-time position of the coil with respect to the head and the estimated induced electric field distribution at the cortical level.

First, the resting motor threshold (RMT) was defined by measuring the motor threshold of the contralateral abductor pollicis brevis (APB) muscle stimulating the primary motor cortex using a coil positioned tangentially to the skull before nrTMS language mapping. RMT was determined for muscles as the minimum stimulation intensity (%) that evoked a clearly distinguishable motor evoked potential (MEP). Each MEP was verified using customized MATLAB software allowing semi-automatic rejection for pre-activation. The ground electrode was placed over the contralateral hand area (abductor pollicis brevis muscle)^24,44,45^. A rough mapping was then started with MEPs between 100 and 500 μV. The RMT was defined as the lowest stimulus intensity sufficient to elicit five MEPs of ≧50 μV in a series of 10 stimuli^46,47^. Motor hotspots were identified with the aid of the anatomical MRI template-guided hardware and software Visor 2 (ANT Neuro, Enschede, the Netherlands). The obtained RMT was used to choose the appropriate intensity for subsequent language mapping while assuring compliance with the risk and safety guidelines^48–50^.

For the language mapping, a picture-naming task was presented on a monitor to identify the cortical language sites using nrTMS. We have used the same picture-naming task for the nrTMS and the DCS language mapping. This task allowed us to check whether patients were able to say aloud the first phrase “this is a …”, to distinguish between speech arrest and anomia^51^. We performed the picture-naming task using a customized LabVIEW program (National Instruments, Austin, TX, USA). Picture presentation on the screen was 700 ms, with an interpicture interval of 2500 ms. To define the appropriate mapping frequency and nrTMS intensity for each patient, a train of 10 TMS bursts was used with 5, 7, or 10 Hz, 10 pulses, and 60–100% RMT. We started nrTMS language mapping at a pulse train of 10 pulses at 5 Hz and were generally able to obtain positive responses. If we could not induce language disruption, we changed the frequency to 5, 7, or 10 Hz. The conditions that were most effective in evoking language errors were then used for language mapping over whole cortical regions. The intensity was increased to 110–120% RMT if there was no apparent effect on picture naming and decreased to 60–90% RMT if patients complained of muscle-related pain or discomfort interfering with the consecutive response evaluation by nrTMS. We sometimes experienced language errors related to muscle stimulation when patients complained of pain or discomfort at the stimulation site. In these cases, we examined all regions of the brain with decreased stimulation intensity. The electric fields induced by the nrTMS parameters ranged between 45 and 80 V/m at the cortical brain surface (the rise time from 0 to peak waveform was between 25.4 and 45.2 A/µs). To ensure the detection of speech arrest and language deficits, the TMS pulse train automatically triggered 300 ms after the picture onset^22,23,52^.

We selected a picture-naming task with black-and-white photographs as the most appropriate cortical stimulation paradigm. A total of 40 photographs of common objects were displayed on a monitor. Before the nrTMS language mapping, the picture-naming task was performed without stimulation to familiarize the patient with the procedure and images. Misnamed pictures were excluded from the stimulation sequence. With the remaining object images, the actual diagnostic naming task was subsequently presented together with a train of nrTMS pulses. The program triggered the onset of every nrTMS pulse train. During the interpicture interval, the stimulation coil was moved to the next cortical stimulation site that was randomly selected. In total, 80–120 sites of the frontal, temporal, and parietal cortex were stimulated three times with nrTMS. In patients with left-side brain tumors, preoperative nrTMS language mapping was only performed on the left side. In contrast, in patients with right-side brain tumors, we mapped both the left and right cerebral cortex. Tumor areas and their proximity were examined in detail.

### Intraoperative DCS language mapping

We performed tumor removal in all 61 patients during awake craniotomy with direct brain stimulation using an asleep-awake-asleep technique, as previously described^11,53–56^. Briefly, the patient underwent craniotomy under general anesthesia. The tumor margins were identified using the neuronavigational system in relation to sulcal and gyral brain surface anatomy, and letter tags were initially placed along the cortical tumor boundaries before the brain shifts.

DCS was applied using a biphasic current (pulse frequency, 60 Hz; single pulse phase duration, 0.5 ms) using a bipolar stimulator with a 2-mm diameter and 5-mm interelectrode distance (Unique Medical, Osaka, Japan). The mapping began at 1 mA, in 0.5-mA increments, until a reproducible functional response was obtained. Thus, the optimal threshold for stimulation was determined and used for the remainder of the cortical and subcortical mapping. Maximum individual current intensities ranged from 2–8 mA. After tumor resection, intraoperative MRI was routinely performed using a 0.4-Tesla vertical field MR scanner (Aperto Inspire; Hitachi, Tokyo, Japan) installed in the operating room of the Brain Theater at Nagoya University Hospital to confirm the EOR of the tumor in all patients^55^.

For the language mapping, a picture-naming task was presented on a monitor to identify the cortical and subcortical language sites using direct electrical stimulation^56,57^. Disruption of object-naming abilities can lead to several types of language deficits, including speech arrest, anomia, dysarthria, anarthria, speech slowness, initiation trouble, perseveration, and paraphasia. This task was performed by asking patients to first test the phrase “this is a …” in order to distinguish speech arrest from anomia^51^. All positive mapping sites were marked with number tags to indicate the specific event that was evoked (speech disorders/arrest, motor dysfunctions, and after discharge). Therefore, recordings of positive responses were transferred to the neuro-navigation system with the navigation pointer. Furthermore, tumor resection was accompanied by subcortical stimulation through the resection cavity, based on the information of the fiber tractography for delineation of white matter anatomy during awake surgery. Thus, intraoperative awake functional mapping for the evaluation of brain functions can define functional boundaries in patients with brain tumors.

### Data analysis of nrTMS and DCS language mapping

If we detected nrTMS-induced language errors such as no-response errors, performance errors, hesitations or the difficulty of word recall, neologisms, semantic paraphasias, phonologic paraphasias, and circumlocutions in at least two out of three trials, the stimulation site was judged as language-positive. In cases of other types of language errors induced by the nrTMS mapping, a site was also considered language-positive. Two experienced speech therapists (H.Y, K.K) analyzed these data independently. The cortical stimulation sites were hidden from these two examiners.

**Figure 5:**
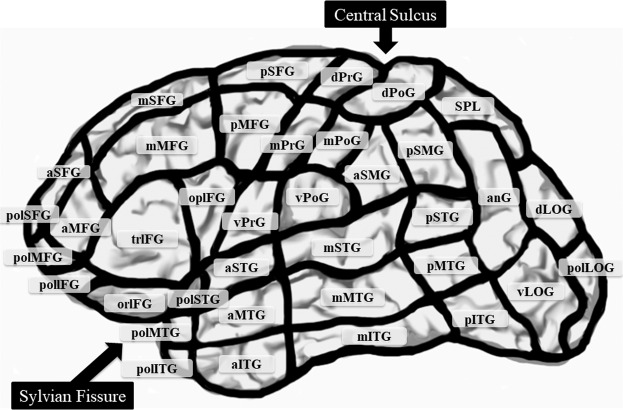
A schematic diagram showing the cortical parcellation system, as described by Corina *et al*.^58^. This schema includes all language-mapped regions: anterior language-related regions (trIFG, opIFG, and vPrG) and posterior language-related regions (aSMG, pSMG, anG, mSTG, and pSTG). All abbreviations are explained in Supplementary Table 1.

To correlate the findings of both nrTMS and intraoperative DCS language mapping at the anatomic level, we assigned them to the language deficits according to CPS^58^ (Fig. 5, Supplementary Table 1). In this method, the cortex is parcellated into 37 individual anatomic brain regions for assessing the anatomic site of the stimulation. Furthermore, we selected anterior language-related CPS regions (trIFG, opIFG, and vPrG), as well as posterior language-related CPS regions (aSMG, pSMG, anG, mSTG, and pSTG; Fig. 5, Supplementary Table 1)^22,24^. Subsequently, the error rate (number of errors per number of stimulations) was calculated for each brain region of the CPS. Two experienced speech therapists (H.Y, K.K) evaluated the language errors for DCS language mapping. Moreover, two independent experienced neurosurgeons (K.M, H.T) assessed the involvement of language areas in the parcellation analysis.

Language positivity or negativity of a CPS region defined with nrTMS was compared with the results of the DCS language mapping^22–24^. When a CPS region was defined as language-positive by both DCS and nrTMS, the region was labeled as a “true positive”. If both DCS and nrTMS indicated a CPS region to be language-negative, the region was regarded as a “true negative”. When nrTMS defined a CPS region as language-positive, but DCS did not, the region was labeled as a “false positive”. Furthermore, a CPS region was documented as a “false negative” when nrTMS did not induce a language error in this region, but DCS determined the region to be language-positive. This procedure was performed separately for each method and for each patient.

### Statistical analysis

ROC curves were used to graphically represent the relationship between sensitivity and specificity for the comparison of nrTMS and DCS language mapping data with respect to language error categories and language-positive or language-negative CPS regions (Fig. 5). Graphs were generated using the R package pROC^59^, which is included in the statistical software R version 3.5.2 (URL: https://www.r-project.org/). AUC values were used to compare TMS performance to DCS data using the method described by DeLong *et al*.^60^, and the 95% confidence intervals (CI) were computed with bootstrap resampling.

We assessed the following subgroups: language-related CPS regions (anterior or posterior), age (≥40 or <40 years), tumor type (high- or low-grade), and tumor volume (≥40 or <40 cm^3^), and the anatomical language-related regions (involvement or non-involvement). Language-related CPS regions: according to CPS, the language mapping area was divided into anterior language-related CPS regions (trIFG, opIFG, and vPrG) or posterior language-related CPS regions (aSMG, pSMG, anG, mSTG, and pSTG; Fig. 5, Supplementary Table 1). Age: median age was 39 years. The age subgroup was divided into big (≥40 years) and small (<40 years). Tumor type: our study included two types of gliomas: 68.9% of low-grade glioma (WHO grade I or II), 31.1% of high-grade glioma (WHO grade III or IV). Median tumor volume was 43.2 cm^3^. Lesions were therefore divided into large (≥40 cm^3^) and small (<40 cm^3^) tumors. Anatomical language-related regions: this subgroup was divided based on whether tumors were located within or without anatomically defined language-eloquent brain regions (anterior or posterior language-related CPS regions).

## Supplementary information

Supplementary information.
