# Supplementary material for: Navigated repetitive transcranial magnetic stimulation as preoperative assessment in patients with brain tumors
Source: Sci Rep. 2020 Jun 3;10:9044. doi: 10.1038/s41598-020-65944-8 (PMC7270124; doi:10.1038/s41598-020-65944-8)
Supplement: Supplementary file 1 — Supplementary information. [file 41598_2020_65944_MOESM1_ESM.docx]

**Navigated repetitive transcranial magnetic stimulation as preoperative assessment in patients with brain tumors**

Kazuya Motomura M.D., Ph.D.^1^*, Hiroki Takeuchi M.D., Ph.D.^2^, Ippei Nojima P.T., Ph.D.^3^,

Kosuke Aoki M.D., Ph.D. ^1^, Lushun Chalise M.D., Ph.D.^1^, Kentaro Iijima M.D., Ph.D.^1^,

Toshihiko Wakabayashi M.D., Ph.D.^1^, Atsushi Natsume M.D., Ph.D.^1^

^1^Department of Neurosurgery, Nagoya University School of Medicine, Nagoya, Japan

^2^Department of Neurosurgery, Higashinagoya National Hospital, Nagoya, Japan

^3^Department of Physical Therapy, Shinshu University School of Medicine, Nagano, Japan

Running title: nrTMS as a preoperative assessment in patients with brain tumors

*Correspondence to:

Kazuya Motomura, M.D., Ph.D.

Department of Neurosurgery, Nagoya University School of Medicine

65 Tsurumai-cho, Showa-ku, Nagoya 466-8550, Japan

Telephone: 81-52-744-2353; Fax: 81-52-744-2360

E-mail: [kmotomura@med.nagoya-u.ac.jp](mailto:kmotomura@med.nagoya-u.ac.jp)

Supplementary Table 1. Abbreviations of the anatomical brain areas in cortical parcellation system

| Abbreviation | Anatomy |
| --- | --- |
| aITG | Anterior inferior temporal gyrus |
| aMFG | Anterior middle frontal gyrus |
| aMTG | Anterior middle temporal gyrus |
| anG | Angular gyrus |
| aSFG | Anterior superior frontal gyrus |
| aSMG | Anterior supramarginal gyrus |
| aSTG | Anterior superior temporal gyrus |
| dLOG | Dorsal lateral occipital gyrus |
| dPoG | Dorsal postcentral gyrus |
| dPrG | Dorsal precentral gyrus |
| mITG | Middle inferior temporal gyrus |
| mMFG | Middle middle frontal gyrus |
| mMTG | Middle middle temporal gyrus |
| mPoG | Middle postcentral gyrus |
| mPrG | Middle precentral gyrus |
| mSFG | Middle superior frontal gyrus |
| mSTG | Middle superior temporal gyrus |
| opIFG | Opercular inferior frontal gyrus |
| orIFG | Orbital part of the inferior frontal gyrus |
| pITG | Posterior inferior temporal gyrus |
| pMFG | Posterior middle frontal gyrus |
| pMTG | Posterior middle temporal gyrus |
| polIFG | Polar inferior frontal gyrus |
| polITG | Polar inferior temporal gyrus |
| polLOG | Polar lateral occipital gyrus |
| polMFG | Polar middle frontal gyrus |
| polMTG | Polar middle temporal gyrus |
| polSFG | Polar superior frontal gyrus |
| polSTG | Polar superior temporal gyrus |
| pSFG | Posterior superior frontal gyrus |
| pSMG | Posterior supramarginal gyrus |
| pSTG | Posterior superior temporal gyrus |
| SPL | Superior parietal lobe |
| trIFG | Triangular inferior frontal gyrus |
| vLOG | Ventral lateral occipital gyrus |
| vPoG | Ventral postcentral gyrus |
| vPrG | Ventral precentral gyrus |
